# Correlation of Ankle-Brachial Index With NT-proBNP and Left Ventricular Ejection Fraction in Patients With Type 2 Diabetes Mellitus and Heart Failure: A Cross-Sectional Study

**DOI:** 10.7759/cureus.115368

**Published:** 2026-08-28

**Authors:** Apoorav Mahajan, Shubhransu Patro, Sambit Das, Anupam Jena, Prithviraj U Naik, Aman Garg, Jyoti Prakash Sahoo

**Affiliations:** 1 General Medicine, Kalinga Institute of Medical Sciences (KIMS), Bhubaneswar, IND; 2 Endocrinology, Kalinga Institute of Medical Sciences (KIMS), Bhubaneswar, IND; 3 Cardiology, Kalinga Institute of Medical Sciences (KIMS), Bhubaneswar, IND; 4 Pharmacology, Kalinga Institute of Medical Sciences (KIMS), Bhubaneswar, IND

**Keywords:** ankle-brachial index (abi), heart failure outcomes, left ventricular ejection fraction (lvef), natriuretic peptide system, new york heart association (nyha), nt-probnp (n terminal-pro-b-type natriuretic peptide), peripheral artery disease (pad), renal impairment, tissue perfusion, type 2 diabetes mellitus (dm)

## Abstract

Background and objectives: Coexistence of type 2 diabetes mellitus (T2DM) and heart failure increases the risk of morbidity and mortality. Ventricular dysfunction and lower limb ischemic changes are assessed with left ventricular ejection fraction (LVEF), NT-proBNP, and ankle-brachial index (ABI). We conducted this study to determine the correlation of ABI with NT-proBNP and LVEF.

Methods: We conducted this cross-sectional study from March 2024 to February 2026 at the Kalinga Institute of Medical Sciences (KIMS), Bhubaneswar, Odisha, India. We enrolled adult diabetic patients with newly diagnosed heart failure. We categorized the participants according to their LVEF: heart failure with preserved ejection fraction (HFpEF) (LVEF ≥ 50%), heart failure with mid-range ejection fraction HFmrEF (LVEF 41%-49%), and heart failure with reduced ejection fraction (HFrEF) (LVEF ≤ 40%). These groups were assessed for ABI and NT-proBNP. We assessed the correlations of ABI with NT-proBNP and of LVEF with Spearman’s correlation. The R software version 4.6.1 (R Foundation for Statistical Computing, Vienna, AUT) was used for data analysis.

Results: Our study population comprised 801 patients (median age 64.0 (59.0-70.0) years; males 447 (55.81%)) with T2DM and heart failure. There were 166 (20.72%), 418 (52.19%), and 217 (27.09%) participants with HFpEF, HFmrEF, and HFrEF, respectively. The median LVEF was 44.0 (39.4-48.8)%. The median ABI of the study population was 1.06 (0.97-1.20). The median ABI values of those with HFpEF, HFmrEF, and HFrEF were 1.04 (0.98-1.17), 1.08 (0.99-1.22), and 1.05 (0.95-1.15), respectively (p < 0.001). The study population’s median NT-proBNP was 1291.0 (978.0-1822.0) pg/mL. The median NT-proBNP values of those with HFpEF, HFmrEF, and HFrEF were 1007.5 (817.0-1370.5) pg/mL, 1295.5 (995.0-1752.3) pg/mL, and 1510.0 (1167.0-2098.0) pg/mL, respectively (p < 0.001). The correlation between ABI and NT-proBNP values was weakly negative (r = -0.059, 95% CI = -0.129 to 0.009, p = 0.139). The correlation between ABI and LVEF was weakly positive (r = 0.057, 95% CI = -0.012 to 0.126, p = 0.293). Only the participants with HFpEF showed a statistically significant positive correlation between ABI and LVEF (r = 0.164, 95% CI = 0.012 to 0.309, p = 0.015). None of the remaining correlations were statistically significant.

Conclusion: Most of the correlations of ABI with NT-proBNP and LVEF were not statistically significant. However, reduced LVEF and higher NT-proBNP levels were correlated with lower ABI values. These findings suggest the associations among elevated cardiac strain, impaired ventricular performance, and peripheral vascular disease. Our study highlighted the usefulness of ABI as a simple, non-invasive screening technique for cardiovascular disease risk.

## Introduction

Type 2 diabetes mellitus (T2DM), the silent epidemic, has encroached every corner of the globe [1]. Being the 'diabetes capital' of the world, India withstands huge socioeconomic and healthcare burdens due to diabetes-related morbidity and mortality [2,3]. Chronic hyperglycemia, along with insulin resistance, advanced glycation end-product (AGE) deposition, oxidative stress, endothelial dysfunction, and inflammatory activation, worsens the functional and structural integrity of the vascular and cardiac tissues [4,5]. Increased arterial stiffness and reduced cardiac perfusion might result from glycation of the extracellular matrix (ECM) proteins in the vasculature and their irreversible cross-linking [5-7]. The AGEs can worsen left ventricular systolic function by accelerating arteriosclerosis [5,6]. Furthermore, AGEs increase the atherogenicity of low-density lipoproteins (LDL) by increasing cross-linking and reducing their clearance [6]. Cardiac contractility is reduced by lowering intracellular calcium [4,6]. These events lead to myocardial rigidity and reduced ECM flexibility [6,7]. Thus, AGE accumulation triggers diastolic dysfunction [5-7].

Heart failure can be functionally classified by the New York Heart Association (NYHA) classification based on the physician’s opinion about the symptoms [8]. Based on the left ventricular ejection fraction (LVEF), heart failure can be categorized as follows: heart failure with preserved ejection fraction (HFpEF) with LVEF ≥ 50%, heart failure with mid-range ejection fraction (HFmrEF) with LVEF 41%-49%, and heart failure with reduced ejection fraction (HFrEF), i.e., LVEF ≤ 40% [9]. Cardiac stress is gauged by natriuretic peptides [10,11]. Both BNP and NT-proBNP are crucial for blood pressure management, lipolysis, and electrolyte and water balance [10,12]. Both are produced in response to neurohormonal stimulation (i.e., the release of angiotensin II and noradrenaline) and mechanical stress [12]. In clinical practice, BNP and NT-proBNP are frequently used to classify patients by risk and to identify those with acute and chronic heart failure [9,12].

The levels of BNP and NT-proBNP generally increase with age [13,14]. Declining renal function is linked to increased BNP and NT-proBNP levels, which render renal failure a risk factor for heart failure [12,14]. The BNP and NT-proBNP are physiologically active and inactive peptides, respectively [12]. The latter has a longer half-life and higher plasma concentrations due to distinct elimination mechanisms [12,15]. Furthermore, NT-proBNP is easier to quantify in routine clinical practice due to its stability [12]. NT-proBNP level is indirectly related to the left ventricle’s function [16]. Plasma NT-proBNP (> 600 pg/mL) indicates left ventricular restricted filling [16]. The normal ranges of NT-proBNP values for adults below and above 75 years are < 125 pg/mL and < 450 pg/mL, respectively [17,18]. In heart failure patients, plasma NT-proBNP is often correlated with E/e', an echocardiographic measure of left ventricular filling pressure [16,19]. Heart failure patients from high-risk groups, such as those with T2DM and/or those who can be identified by general practitioners by assessing NT-proBNP level [16-19]. This might expedite medication uptitration or the use of innovative therapies, thereby delaying the progression of heart failure [16,18,19].

Patients with heart failure and peripheral arterial disease (PAD) experience worse clinical outcomes in contrast to those without PAD [20]. The prevalence of PAD in heart failure patients seems to be underestimated because roughly half of patients with PAD are asymptomatic [20,21]. Ankle-brachial index (ABI) testing is a noninvasive diagnostic tool that identifies patients with heart failure who may have worse outcomes [20,22]. Its normal value ranges between 0.9 and 1.3 [22-24]. General practitioners should check for PAD when a patient’s ABI falls below 0.9 [23,24]. The ABI is a predictive indicator for vascular impairment even in the absence of PAD symptoms [22,23]. Arteriosclerosis and arterial calcification are more common in the lower than in the upper limbs [23]. The ABI helps detect lower-extremity ischemic disease [22,23]. There are limited studies regarding ABI, NT-proBNP, and LVEF in patients with T2DM and heart failure. Hence, we conducted this study to determine the correlations between ABI, NT-proBNP, and LVEF in patients with T2DM and heart failure.

## Materials and methods

Study design and criteria

We conducted this cross-sectional study at the Kalinga Institute of Medical Sciences (KIMS), Bhubaneswar, India, from March 2024 to February 2026. Before commencing the study, we obtained approval from the Institutional Ethics Committee (approval no. KIIT/KIMS/IEC/1527/2024 dated 06.02.2024). We screened all adult patients with T2DM and newly diagnosed heart failure. The endocrinologist (our co-investigator) suspected that some of the adult diabetic patients had heart disease and therefore referred them for a cardiology consultation. Of these referred patients, we enrolled those with newly diagnosed heart failure (confirmed by our co-investigator cardiologist on their prescription slips). We excluded patients with any congenital or valvular heart disease, arrhythmia, myocardial ischemia, infarction, or diabetes mellitus other than type 2. Patients on any pharmacotherapy for heart failure or with amputated limbs were also excluded.

Study procedure

After participant enrollment, we retrieved demographic details, including age, sex, and socioeconomic status, from their case record sheets. The Kuppuswamy classification [25] was used to classify socioeconomic status. We also checked whether anyone had hypertension, obesity, dyslipidemia, or thyroid dysfunction. We could only collect data regarding hypertension and obesity. We used body mass index (BMI) to assess obesity. Most of the participants did not have recent thyroid function test and fasting lipid profile reports, nor did they agree to have the tests done. Therefore, we analyzed BMI and the presence of hypertension among the participants. We had the following parameters for all participants: fasting blood sugar (FBS), 2-hour postprandial blood sugar (PPBS), glycated hemoglobin (HbA1c), and serum creatinine. We computed the estimated glomerular filtration rate (eGFR) using the Modification of Diet in Renal Disease (MDRD) formula [26] and categorized them by eGFR.

All of them were tested for ABI, ECG, LVEF, and NT-proBNP levels as per the cardiologist’s advice. For ABI measurement, the participants were advised to avoid smoking, alcohol, caffeine, and exercise one hour before the test. We used a mercury sphygmomanometer to check the bilateral ankle and brachial blood pressures. The higher values of ankle and brachial blood pressures were noted. The ABI was computed by dividing the higher ankle pressure by the higher brachial pressure. We also measured ABI through the Dopplex® Ability Automatic ABI System (Huntleigh Healthcare Ltd., Cardiff, GBR). For each participant, we got two different ABIs (one from manual calculation and another from the automatic device). We noted the higher value for the analysis.

On each participant’s prescription, the cardiologist had mentioned their NYHA functional class and type of heart failure based on their LVEF. Left ventricular contractility is indicated by LVEF and depends on the degree of change in LV volume from ventricular diastole to ventricular systole. Stroke volume (SV) is the difference between end-diastolic LV volume (EDV) and end-systolic LV volume (ESV). The LVEF is mathematically calculated by dividing SV by EDV. This method is the simplest and widely accepted by cardiologists. During echocardiography, the cardiologist measured EDV, ESV, SV, and LVEF. We noted those values for all participants. The LVEF varies with age, sex, race, comorbidities, and measurement method. As per the heart’s pumping efficiency, the types of heart failure are as follows: HFpEF (LVEF: ≥ 50%), HFmrEF (LVEF: 41%-49%), and HFrEF (LVEF: ≤ 40%).

For the quantification of NT-proBNP, we used the Cobas® e411 analyzer (Roche Diagnostics, Basel, CHE). The manufacturer's reagents were used for calibration and quality control purposes. The manufacturer sets the NT-proBNP assay's limit of detection at 10 pg/mL. Single thaws of serum aliquots were used to determine the NT-proBNP levels of the participants.

Statistical analysis

We recruited all eligible patients during the above-mentioned duration through non-probability consecutive sampling. We assessed the normality of the data distribution using the Kolmogorov-Smirnov test. We analyzed all parameters (i.e., age, sex, BMI, serum creatinine, eGFR, LVEF, ABI, and NT-proBNP) across participants with different types of heart failure, i.e., HFpEF, HFmrEF, and HFrEF. We reported continuous data in our study as medians with IQRs and categorical data as numbers with percentages. For the continuous data, we used the Kruskal-Wallis test and computed the H-values. For the categorical data, we used the chi-square test and computed the chi-square values. We illustrated the study population's distribution through a mosaic plot. For the illustration of quantitative data, we used box-and-whisker, jitter, and violin plots. We used Spearman’s correlation to assess the correlations of ABI with NT-proBNP and LVEF. The correlation coefficients (r) were calculated with 95% CI and p-values. The correlations were shown through two different scatter plots. For the correlation between ABI and NT-proBNP, we used three different colors to denote the participants with HFpEF, HFmrEF, and HFrEF. However, we portrayed the correlation between ABI and LVEF in a single color. For data analysis, we used R software version 4.6.1(R Foundation for Statistical Computing, Vienna, AUT) [27]. Statistical significance was achieved with p-values < 0.05.

## Results

Overview of the study population

During our study period, 2468 patients who visited the cardiology OPD had heart failure. Of these, 739 (29.94%) patients were already on pharmacotherapy for heart failure. The blood reports and clinical assessments of 928 patients (37.60%) supported their non-diabetic status. The remaining 801 (32.46%) patients were enrolled in this study. Table 1 demonstrates the demographic and clinical details of these 801 participants. Of them, 447 (55.81%) were males. Among the enrolled patients, HFpEF was observed in 166 (20.72%), HFmrEF in 418 (52.19%), and HFrEF in 217 (27.09%) participants, respectively; and 518 (64.67%) participants were on antihypertensive medications. The median LVEF was 44.0% (39.4-48.8). The median values of serum creatinine and eGFR were 1.11 (0.89-1.43) mg/dL and 56.4 (40.6-70.9) mL/min/1.73 m², respectively.

**Table 1:** Demographic and clinical traits of the study participants (total n = 801) The continuous and categorical variables were presented as medians with IQRs and frequencies with proportions, respectively. We used the Kruskal-Wallis test for the continuous variables and computed H-values. For the categorical variables, we used the chi-square test and computed the chi-square values. HFpEF: Heart failure with preserved ejection fraction, HFmrEF: Heart failure with mid-range ejection fraction, HFrEF: Heart failure with reduced ejection fraction, LVEF: Left ventricular ejection fraction, NYHA: The New York Heart Association, eGFR: Estimated glomerular filtration rate

Parameter		Total (n = 801)	HFpEF (n = 166)	HFmrEF (n = 418)	HFrEF (n = 217)	Statistical test used	Test statistics	p-value
Age	Age (years)	64.0 (59.0-70.0)	64.0 (60.0-69.0)	64.0 (59.0-69.0)	66.0 (59.0-71.0)	Kruskal-Wallis test	3.41	0.182
	Age > 65 years	365 (45.57%)	76 (45.78%)	175 (41.87%)	114 (52.53%)	Chi-square test	6.559	0.038
Gender	Male patients	447 (55.81%)	94 (56.63%)	230 (55.02%)	123 (56.68%)	Chi-square test	0.217	0.897
Socioeconomic class	Lower	141 (17.60%)	32 (19.28%)	66 (15.79%)	43 (19.82%)	Chi-square test	474.37	< 0.001
	Lower middle	428 (53.43%)	103 (62.05%)	271 (64.83%)	54 (24.88%)			
	Upper middle	232 (28.96%)	31 (18.67%)	81 (19.38%)	120 (55.30%)			
BMI category	BMI (kg/m^2^)	27.7 (26.1-29.7)	26.7 (25.6-29.7)	28.0 (26.3-30.0)	28.0 (26.8-29.6)	Kruskal-Wallis test	11.15	0.004
	Normal weight (18.5-24.9 kg/m^2^)	34 (4.24%)	8 (4.82%)	16 (3.83%)	10 (4.61%)	Chi-square test	35.91	< 0.001
	Overweight (25.0-29.9 kg/m^2^)	572 (71.41%)	119 (71.69%)	297 (71.05%)	156 (71.89%)			
	Obesity (≥ 30 kg/m^2^)	195 (24.34%)	39 (23.49%)	105 (25.12%)	51 (23.50%)			
	Hypertension	518 (64.67%)	81 (48.80%)	258 (61.72%)	179 (82.49%)	Chi-square test	442.81	< 0.001
NYHA class	I	124 (15.48%)	117 (70.48%)	7 (1.67%)	0	Chi-square test	501.06	< 0.001
	II	284 (35.46%)	49 (29.52%)	227 (54.31%)	8 (3.69%)			
	III	313 (39.08%)	0	184 (44.02%)	129 (59.44%)			
	IV	80 (9.99%)	0	0	80 (36.87%)			
	LVEF (%)	44.0 (39.4-48.8)	53.7 (51.1-56.4)	44.5 (42.3-47.3)	37.0 (34.2-38.6)	Kruskal-Wallis test	663.34	< 0.001
	Serum creatinine (mg/dL)	1.11 (0.89-1.43)	1.02 (0.86-1.27)	1.09 (0.91-1.49)	1.13 (0.96-1.57)	Kruskal-Wallis test	15.326	< 0.001
	eGFR (mL/min/1.73 m^2^)	56.4 (40.6-70.9)	62.9 (52.5-75.1)	57.3 (39.7-72.4)	51.2 (36.6-68.3)	Kruskal-Wallis test	19.149	< 0.001
eGFR stage	G2 (60-89 mL/min/1.73 m^2^)	381 (47.57%)	94 (56.63%)	193 (46.17%)	94 (43.32%)	Chi-square test	77.987	< 0.001
	G3 (30-59 mL/min/1.73 m^2^)	307 (38.33%)	59 (35.54%)	164 (39.23%)	84 (38.71%)			
	G4 (15-29 mL/min/1.73 m^2^)	113 (14.11%)	13 (7.83%)	61 (14.59%)	39 (17.97%)			

**Figure 1:**
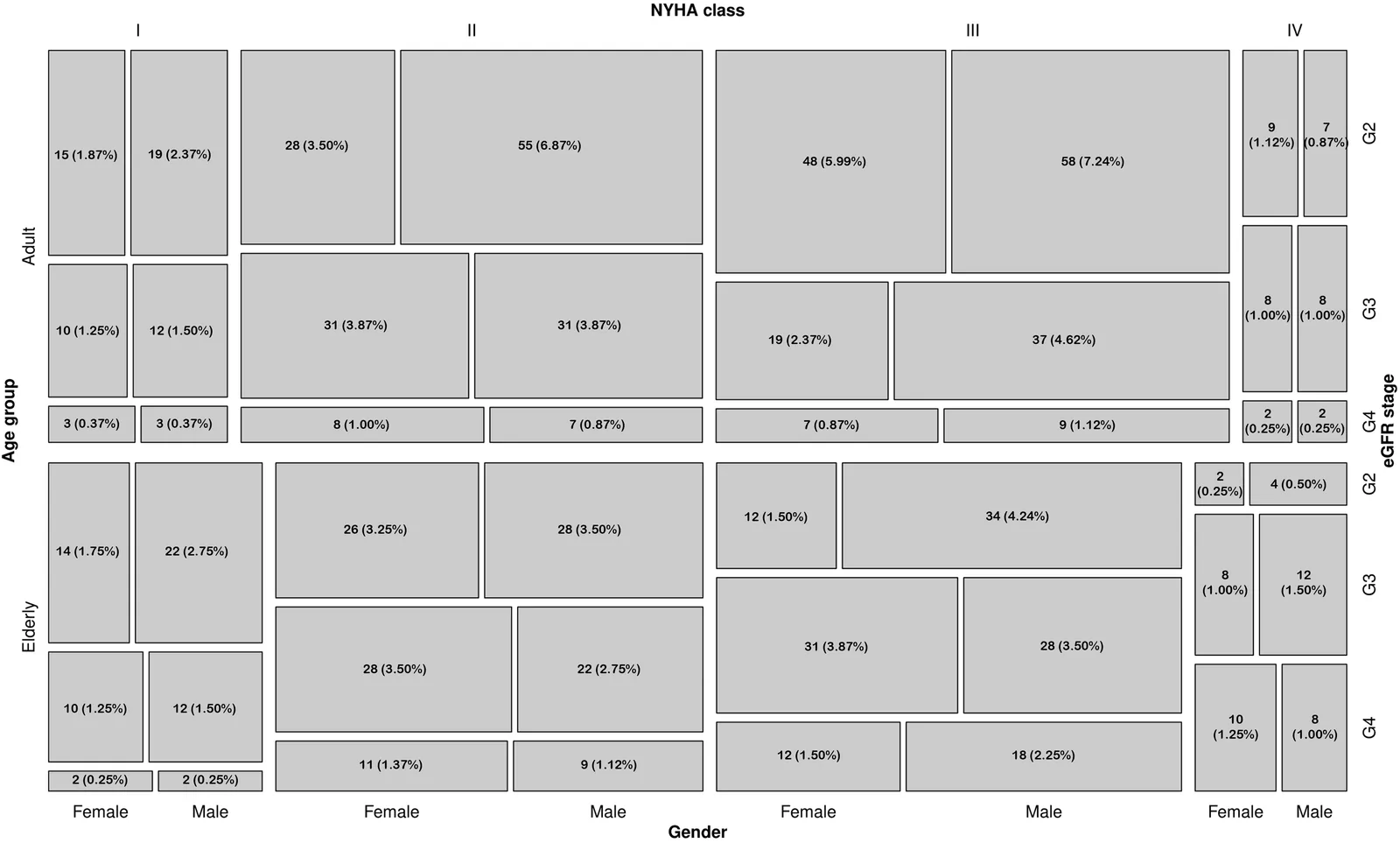
Distribution of the study population The mosaic plot illustrates the distribution of participants by age group (adult or elderly), NYHA class (I, II, III, or IV), eGFR stage (G2, G3, or G4), and gender (female or male). The authors used R software version 4.6.1 (R Foundation for Statistical Computing, Vienna, AUT) [27] to generate this plot. NYHA: The New York Heart Association, eGFR: Estimated glomerular filtration rate

The mosaic plot in Figure 1 illustrates the distribution of participants by gender, age group, NYHA class for heart failure, and eGFR stage for renal impairment. The categorization order was age group, NYHA class, eGFR stage, and gender. The largest block portrays the adult male patients with NYHA class III and G2 stage (58, 7.24%), followed by the adult male patients with NYHA class II and G2 stage (55, 6.87%), the adult female patients with NYHA class III and G2 stage (48, 5.99%), and the adult male patients with NYHA class III and G3 stage (37, 4.62%).

Age and BMI of participants

**Figure 2:**
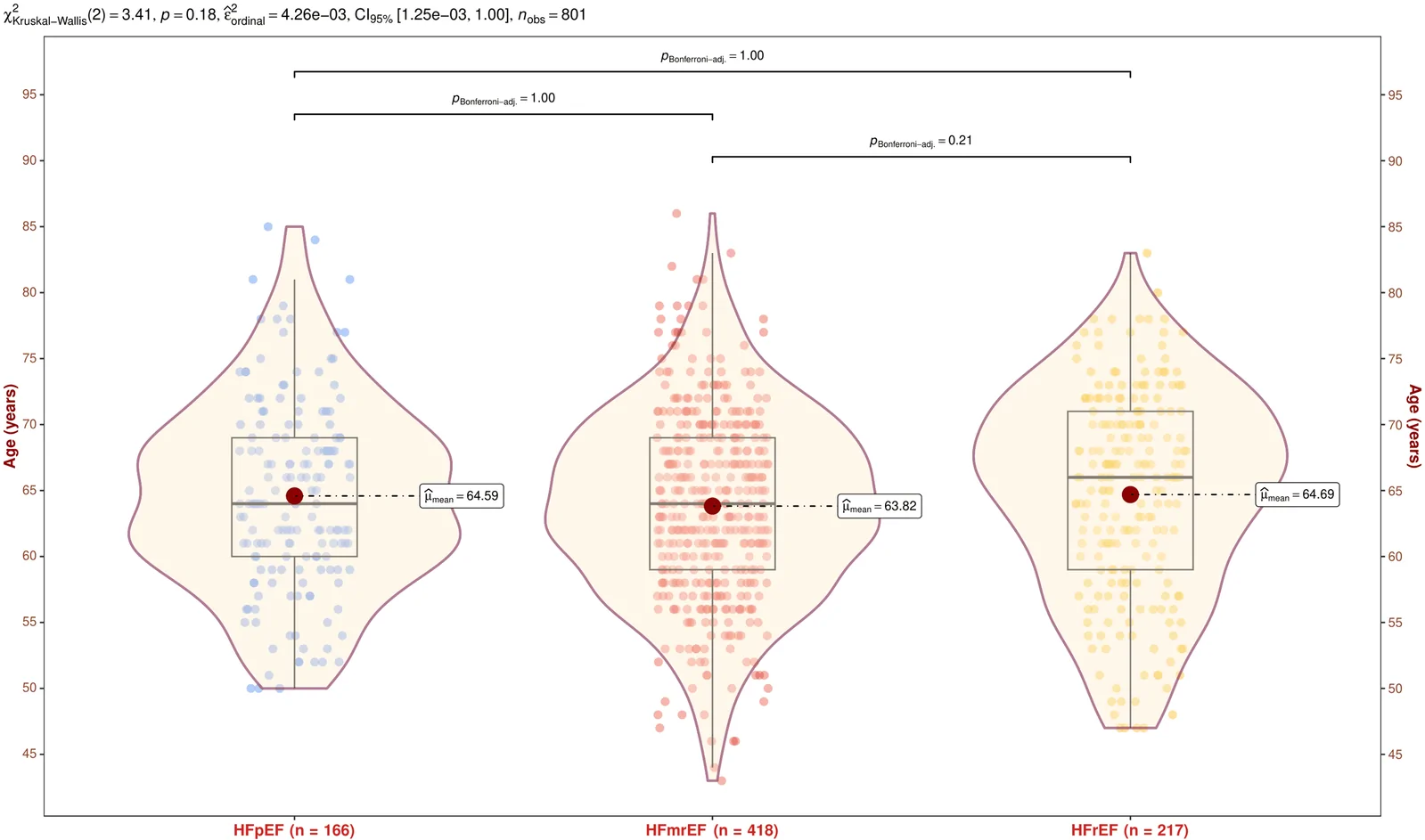
Age distribution of the study participants The box-and-whisker, jitter, and violin plots demonstrate the age of all participants (n = 801). The red dots represent the mean values. We used the Kruskal-Wallis test to analyze these variables and the Bonferroni adjustment for the post hoc analysis. A p-value < 0.05 was deemed statistically significant. The authors used R software version 4.6.1 (R Foundation for Statistical Computing, Vienna, AUT) [27] to generate this plot. HFpEF: Heart failure with preserved ejection fraction, HFmrEF: Heart failure with mid-range ejection fraction, HFrEF: Heart failure with reduced ejection fraction, LVEF: Left ventricular ejection fraction

Figure 2 shows the age distribution of the participants. Their median age was 64.0 (59.0-70.0) years. The median ages of the participants with HFpEF, HFmrEF, and HFrEF were 64.0 (60.0-69.0) years, 64.0 (59.0-69.0) years, and 66.0 (59.0-71.0) years, respectively (p = 0.182). The age group distribution is shown in Table 1.

**Figure 3:**
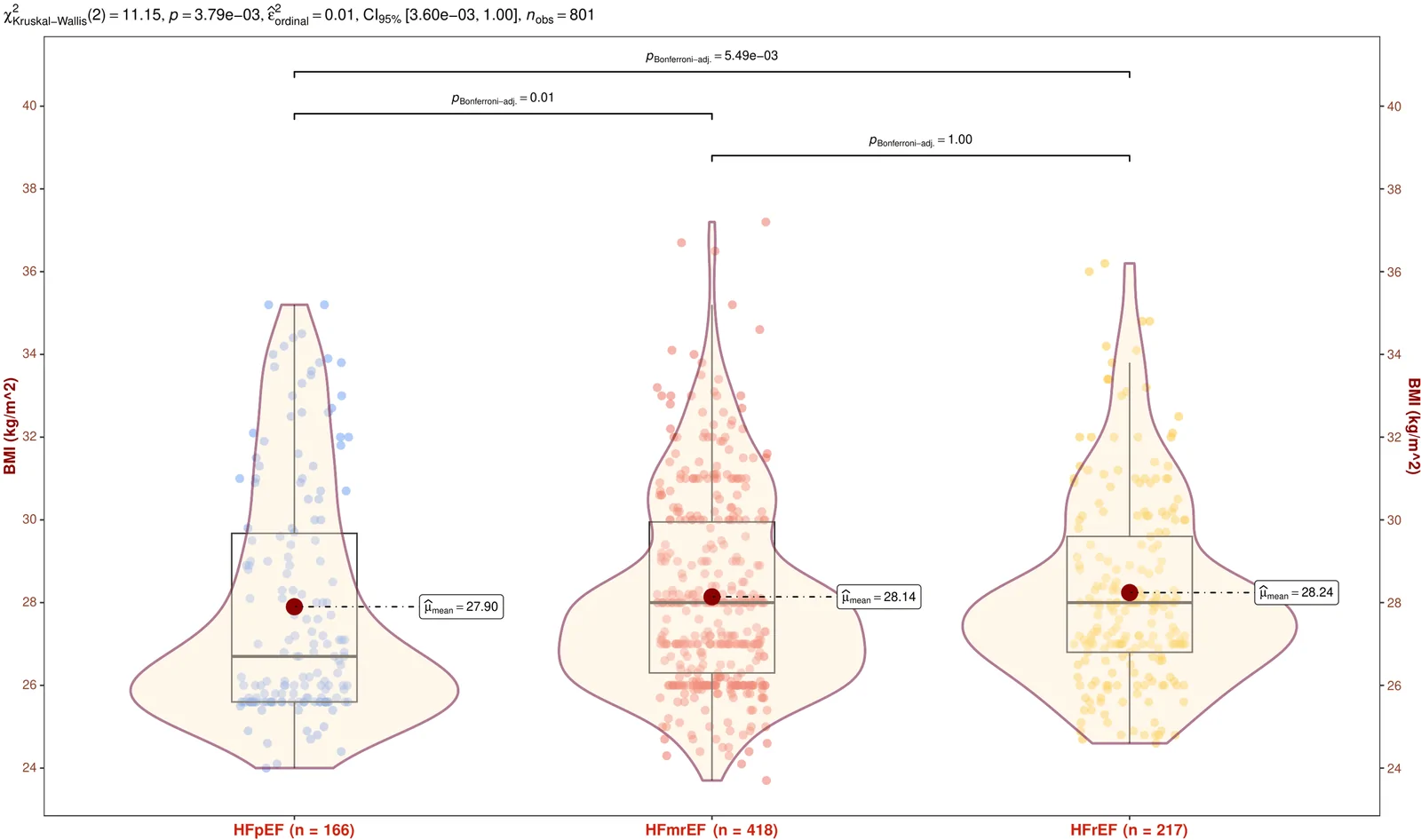
BMI of the study participants The box-and-whisker, jitter, and violin plots demonstrate the BMI of all participants (n = 801). The red dots represent the mean values. We used the Kruskal-Wallis test to analyze these variables and the Bonferroni adjustment for the post hoc analysis. A p-value < 0.05 was deemed statistically significant. The authors used R software version 4.6.1 (R Foundation for Statistical Computing, Vienna, AUT) [27] to generate this plot. HFpEF: Heart failure with preserved ejection fraction, HFmrEF: Heart failure with mid-range ejection fraction, HFrEF: Heart failure with reduced ejection fraction, LVEF: Left ventricular ejection fraction

Figure 3 shows the BMI distribution of the participants. Their median BMI was 27.7 (26.1-29.7) kg/m². The median ages of the participants with HFpEF, HFmrEF, and HFrEF were 26.7 (25.6-29.7) kg/m², 28.0 (26.3-30.0) kg/m², and 28.0 (26.8-29.6) kg/m², respectively (p < 0.001). The BMI categorization of the participants is shown in Table 1.

Glycemic parameters

**Figure 4:**
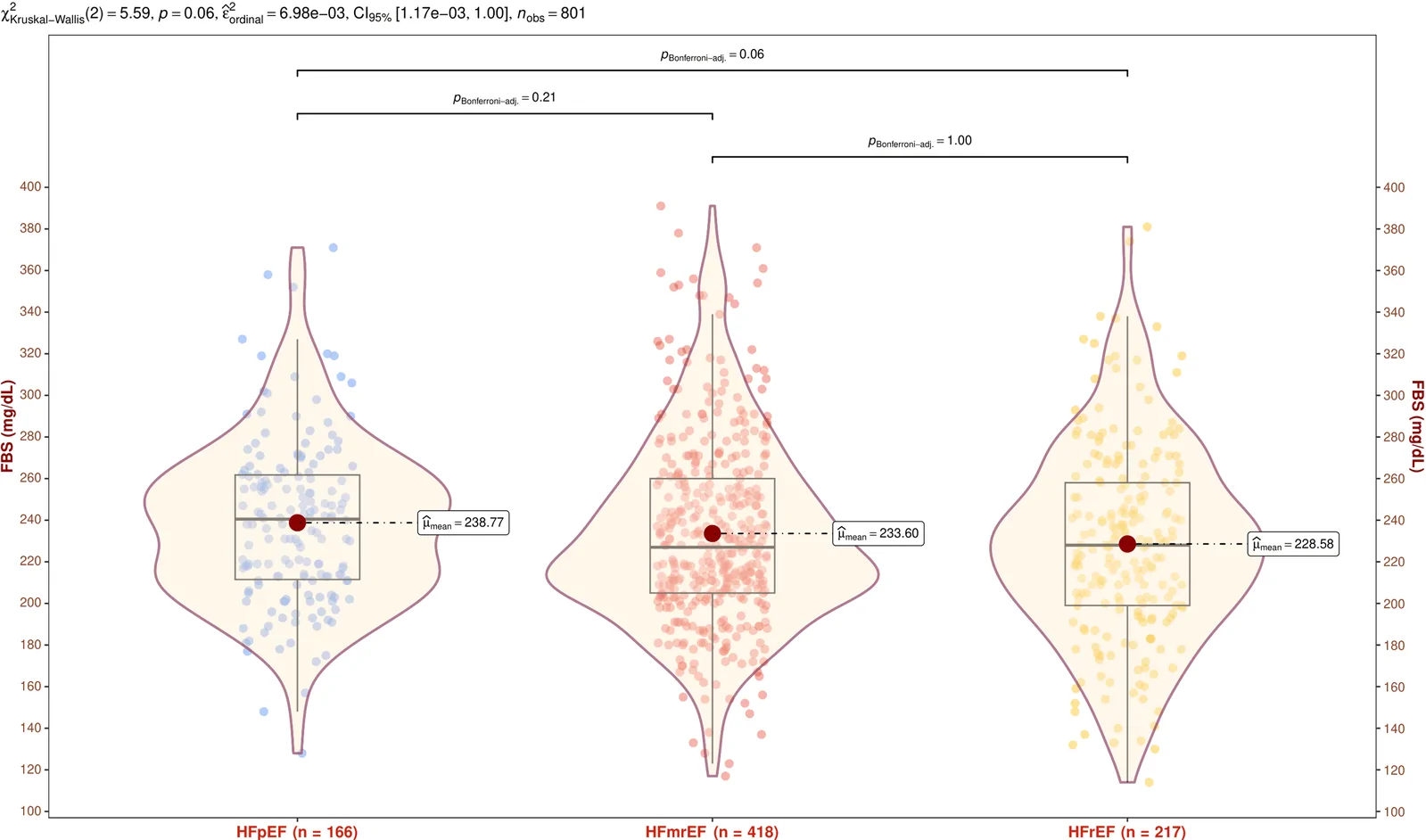
FBS values of the study participants The box-and-whisker, jitter, and violin plots show the FBS for all participants (n = 801). The red dots represent the mean values. We used the Kruskal-Wallis test to analyze these variables and the Bonferroni adjustment for the post hoc analysis. A p-value < 0.05 was deemed statistically significant. The authors used R software version 4.6.1 (R Foundation for Statistical Computing, Vienna, AUT) [27] to generate this plot. HFpEF: Heart failure with preserved ejection fraction, HFmrEF: Heart failure with mid-range ejection fraction, HFrEF: Heart failure with reduced ejection fraction, LVEF: Left ventricular ejection fraction

Figure 4 and Table 2 show the FBS values of the participants. Their median FBS was 230.0 (204.0-260.0) mg/dL. The median FBS values of the participants with HFpEF, HFmrEF, and HFrEF were 240.5 (211.5-261.8) mg/dL, 227.0 (205.0-260.0) mg/dL, and 228.0 (199.0-258.0) mg/dL, respectively (p = 0.06).

**Table 2:** Glycemic parameters of the study participants The FBS, PPBS, and HbA_1c_ were expressed as medians and IQRs. We used the Kruskal-Wallis test to analyze these variables and the Bonferroni adjustment for the post hoc analysis. A p-value < 0.05 was deemed statistically significant. HFpEF: Heart failure with preserved ejection fraction, HFmrEF: Heart failure with mid-range ejection fraction, HFrEF: Heart failure with reduced ejection fraction, LVEF: Left ventricular ejection fraction, FBS: Fasting blood sugar, PPBS: Post-prandial blood sugar, HbA_1c_: Glycated hemoglobin

Parameter	Total (n = 801)	HFpEF (n = 166)	HFmrEF (n = 418)	HFrEF (n = 217)	H-value	p-value
FBS (mg/dL)	230.0 (204.0-260.0)	240.5 (211.5-261.8)	227.0 (205.0-260.0)	228.0 (199.0-258.0)	5.59	0.06
PPBS (mg/dL)	305.0 (268.0-351.0)	306.5 (274.8-352.0)	304.5 (267.0-354.0)	302.0 (262.0-346.0)	1.91	0.39
HbA_1c _(%)	9.10 (8.00-10.55)	9.38 (8.28-10.70)	9.04 (7.96-10.77)	9.01 (7.80-10.17)	7.72	0.02

**Figure 5:**
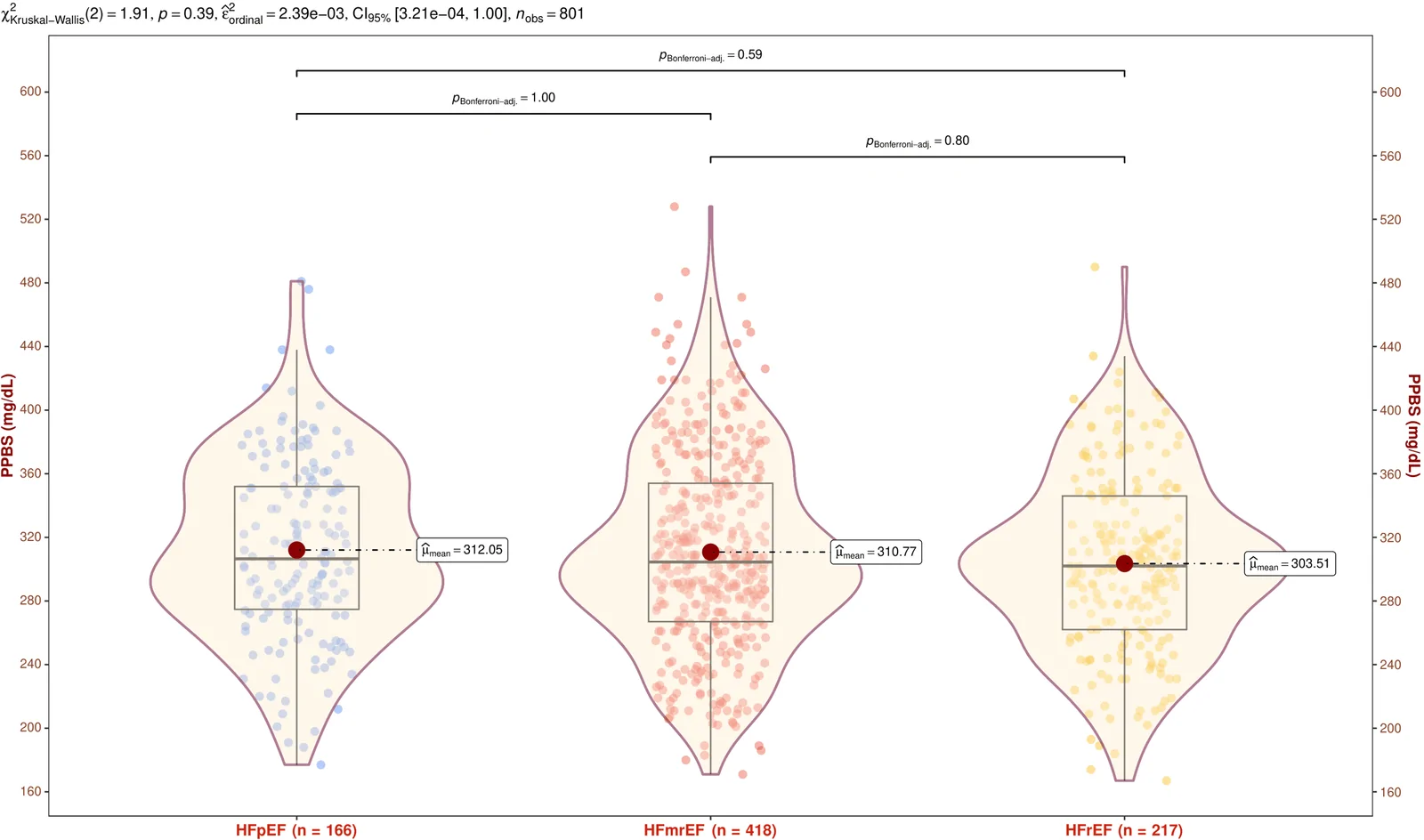
PPBS values of the study participants The box-and-whisker, jitter, and violin plots show the PPBS for all participants (n = 801). The red dots represent the mean values. We used the Kruskal-Wallis test to analyze these variables and the Bonferroni adjustment for the post hoc analysis. A p-value < 0.05 was deemed statistically significant. The authors used R software version 4.6.1 (R Foundation for Statistical Computing, Vienna, AUT) [27] to generate this plot. HFpEF: Heart failure with preserved ejection fraction, HFmrEF: Heart failure with mid-range ejection fraction, HFrEF: Heart failure with reduced ejection fraction, LVEF: Left ventricular ejection fraction, PPBS: Post-prandial blood sugar

Figure 5 and Table 2 show the PPBS values of the participants. Their median PPBS was 305.0 (268.0-351.0) mg/dL. The median PPBS values of the participants with HFpEF, HFmrEF, and HFrEF were 306.5 (274.8-352.0) mg/dL, 304.5 (267.0-354.0) mg/dL, and 302.0 (262.0-346.0) mg/dL, respectively (p = 0.39).

**Figure 6:**
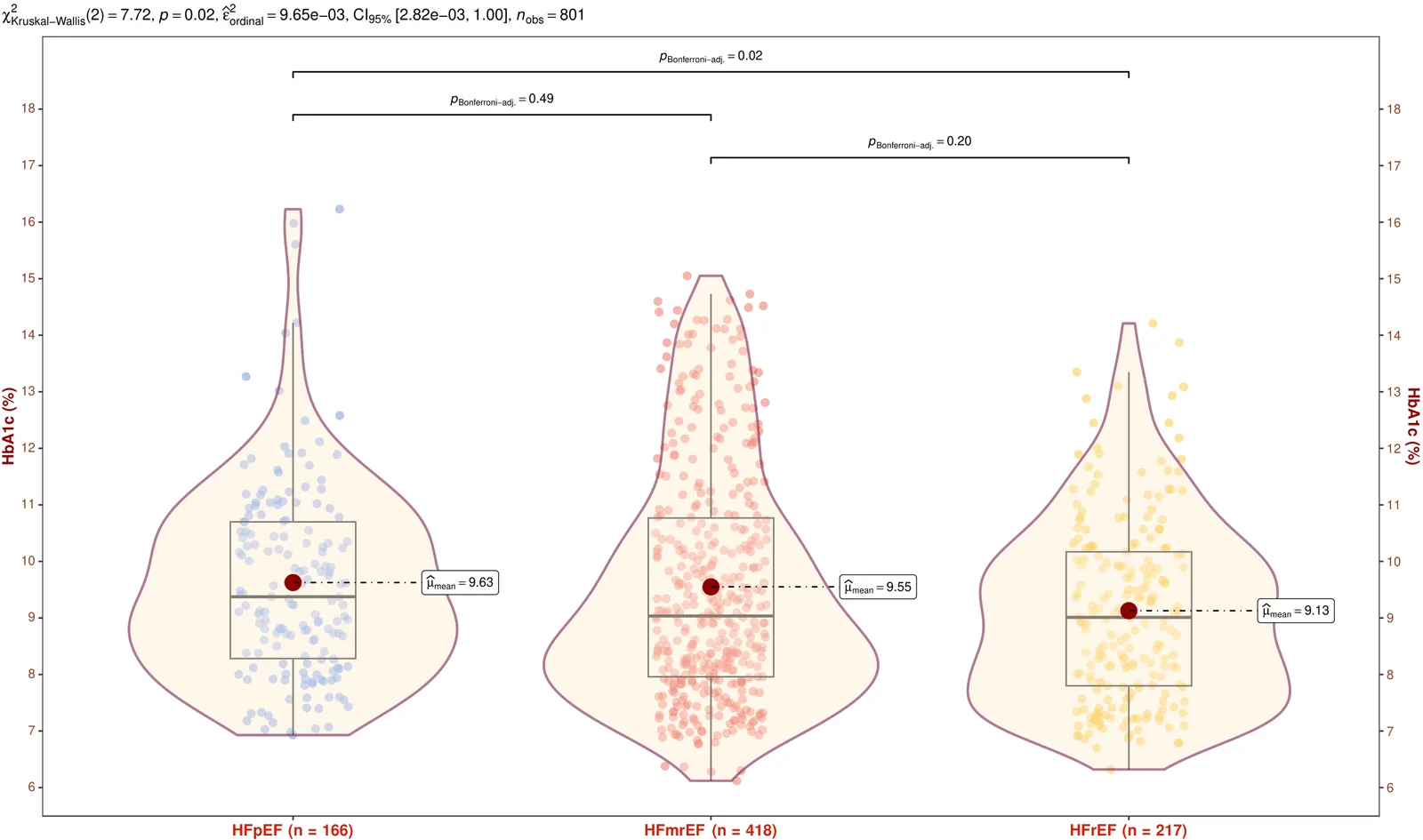
HbA1c values of the study participants The box-and-whisker, jitter, and violin plots demonstrate HbA_1c_ of all participants (n = 801). The red dots represent the mean values. We used the Kruskal-Wallis test to analyze these variables and the Bonferroni adjustment for the post hoc analysis. A p-value < 0.05 was deemed statistically significant.  The authors used R software version 4.6.1 (R Foundation for Statistical Computing, Vienna, AUT) [27] to generate this plot. HFpEF: Heart failure with preserved ejection fraction, HFmrEF: Heart failure with mid-range ejection fraction, HFrEF: Heart failure with reduced ejection fraction, LVEF: Left ventricular ejection fraction, HbA_1c_: Glycated hemoglobin

Figure 6 and Table 2 show the HbA1c values of the study population. Their median HbA1c was 9.10% (8.00-10.55). The median HbA1c values of the participants with HFpEF, HFmrEF, and HFrEF were 9.38% (8.28-10.70), 9.04% (7.96-10.77), and 9.01% (7.80-10.17), respectively (p = 0.02).

ABI and NT-proBNP values of participants

**Figure 7:**
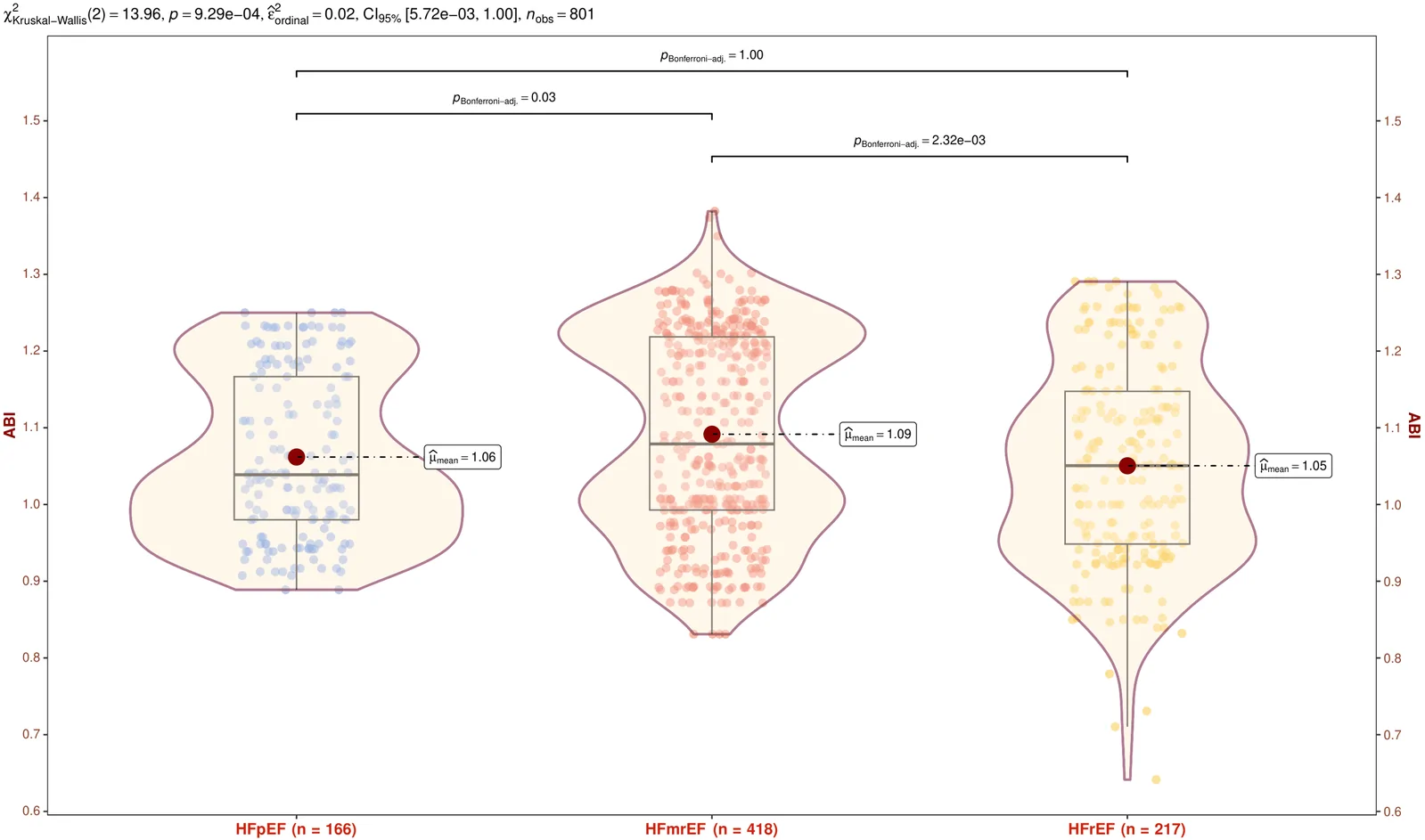
ABI values of the study participants The box-and-whisker, jitter, and violin plots show the ABI for all participants (n = 801). The red dots represent the mean values. The normal range of ABI is 0.9-1.3. We used the Kruskal-Wallis test to analyze these variables and the Bonferroni adjustment for the post hoc analysis. A p-value < 0.05 was deemed statistically significant. The authors used R software version 4.6.1 (R Foundation for Statistical Computing, Vienna, AUT) [27] to generate this plot. HFpEF: Heart failure with preserved ejection fraction, HFmrEF: Heart failure with mid-range ejection fraction, HFrEF: Heart failure with reduced ejection fraction, LVEF: Left ventricular ejection fraction, ABI: Ankle-brachial index

Figure 7 and Table 3 show the ABI of the participants. Their median ABI was 1.06 (0.97-1.20). The median ABI values of the participants with HFpEF, HFmrEF, and HFrEF were 1.04 (0.98-1.17), 1.08 (0.99-1.22), and 1.05 (0.95-1.15), respectively (p < 0.001).

**Table 3:** ABI and NT-proBNP values of the study participants The ABI and NT-proBNP values were expressed as medians and IQRs. We used the Kruskal-Wallis test to analyze these variables and the Bonferroni adjustment for the post hoc analysis. The normal range of ABI is 0.9-1.3. The normal ranges of NT-proBNP values for adults below and above 75 years are < 125 pg/mL and < 450 pg/mL, respectively. A p-value < 0.05 was deemed statistically significant. HFpEF: Heart failure with preserved ejection fraction, HFmrEF: Heart failure with mid-range ejection fraction, HFrEF: Heart failure with reduced ejection fraction, LVEF: Left ventricular ejection fraction, ABI: Ankle-brachial index

Parameter	Total (n = 801)	HFpEF (n = 166)	HFmrEF (n = 418)	HFrEF (n = 217)	H-value	p-value
ABI	1.06 (0.97-1.20)	1.04 (0.98-1.17)	1.08 (0.99-1.22)	1.05 (0.95-1.15)	13.96	< 0.001
NT-proBNP (pg/mL)	1291.0 (978.0-1822.0)	1007.5 (817.0-1370.5)	1295.5 (995.0-1752.3)	1510.0 (1167.0-2098.0)	68.44	< 0.001

**Figure 8:**
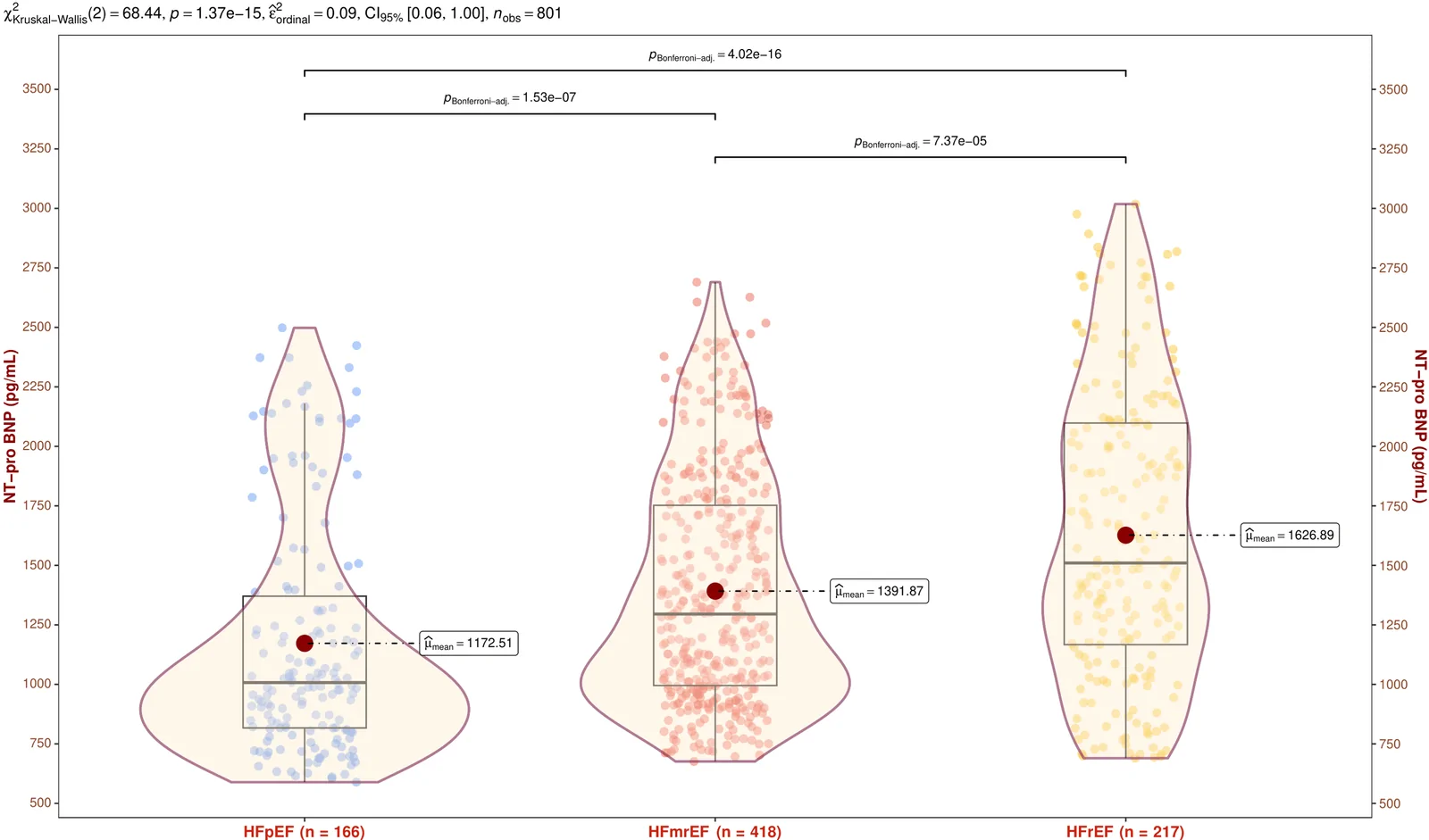
NT-proBNP values of the study participants The box-and-whisker, jitter, and violin plots show NT-proBNP values for all participants (n = 801). The red dots represent the mean values. The normal ranges of NT-proBNP values for adults below and above 75 years are <125 pg/mL and <450 pg/mL, respectively. We used the Kruskal-Wallis test to analyze these variables and the Bonferroni adjustment for the post hoc analysis. A p-value < 0.05 was deemed statistically significant. The authors used R software version 4.6.1 (R Foundation for Statistical Computing, Vienna, AUT) [27] to generate this plot. HFpEF: Heart failure with preserved ejection fraction, HFmrEF: Heart failure with mid-range ejection fraction, HFrEF: Heart failure with reduced ejection fraction, LVEF: Left ventricular ejection fraction

Figure 8 and Table 3 show the NT-proBNP values of the participants. Their median NT-proBNP was 1291.0 (978.0-1822.0) pg/mL. The median NT-proBNP values of the participants with HFpEF, HFmrEF, and HFrEF were 1007.5 (817.0-1370.5) pg/mL, 1295.5 (995.0-1752.3) pg/mL, and 1510.0 (1167.0-2098.0) pg/mL, respectively (p < 0.001).

Correlations of ABI

**Figure 9:**
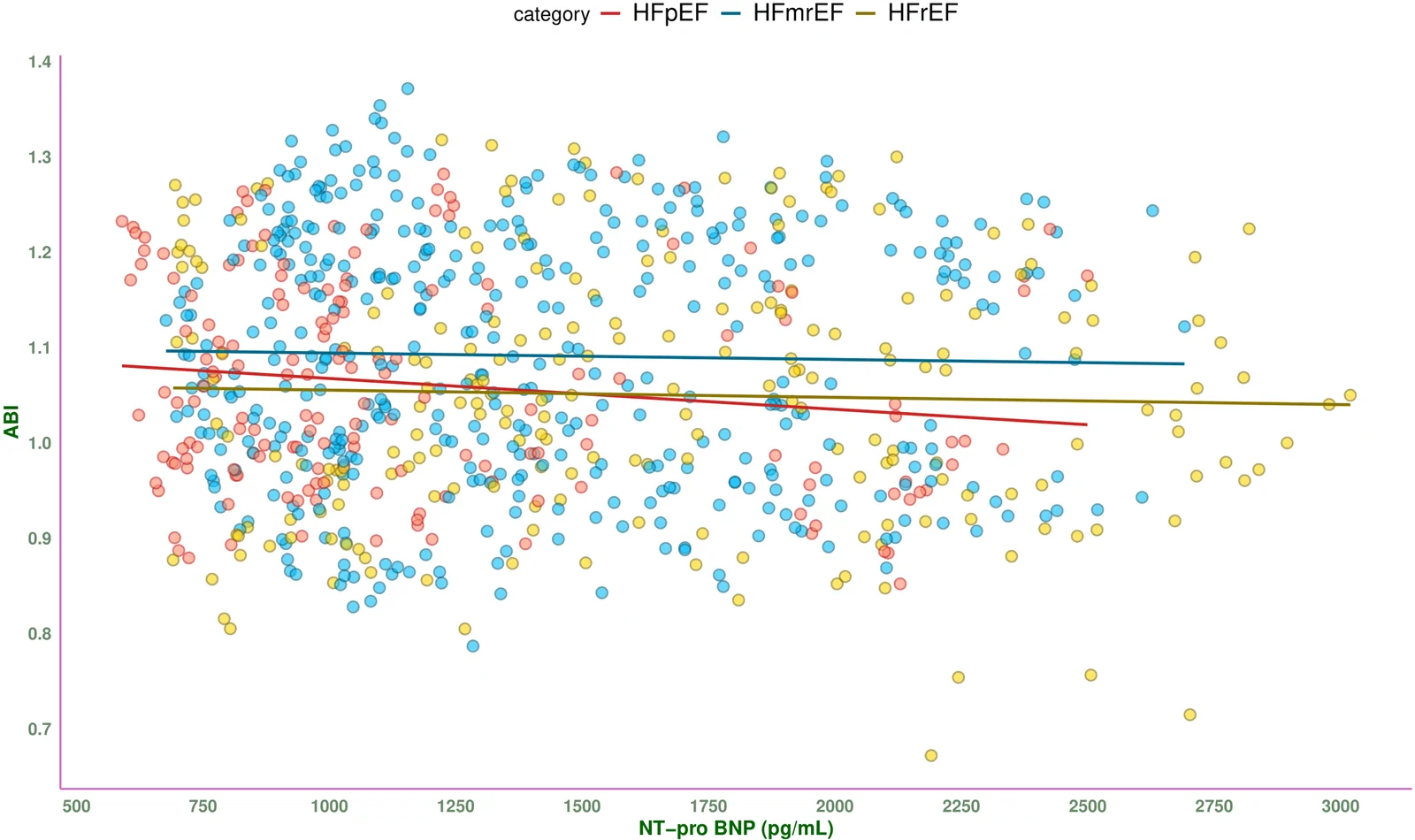
Correlation of ABI and NT-proBNP values The jitter plots illustrate the correlation between ABI and NT-proBNP among the participants. We used Spearman’s correlation to assess the same. A p-value < 0.05 was deemed statistically significant. The authors used R software version 4.6.1 (R Foundation for Statistical Computing, Vienna, AUT) [27] to generate this plot. HFpEF: Heart failure with preserved ejection fraction, HFmrEF: Heart failure with mid-range ejection fraction, HFrEF: Heart failure with reduced ejection fraction, LVEF: Left ventricular ejection fraction, ABI: Ankle-brachial index

Figure 9 and Table 4 demonstrate the correlation between ABI and NT-proBNP values among participants. There was a weak negative correlation (r = -0.059, 95% CI = -0.129 to 0.009, p = 0.139) between the ABI and NT-proBNP values of all participants. The correlations were also negative among those with HFpEF (r = -0.149, 95% CI = -0.294 to 0.004, p = 0.077), HFmrEF (r = -0.025, 95% CI = -0.120 to 0.071, p = 0.359), and HFrEF (r = -0.035, 95% CI = -0.167 to 0.099, p = 0.774).

**Table 4:** Correlations of ABI with NT-proBNP and LVEF We checked the correlations of ABI with NT-proBNP and LVEF of the participants. We used Spearman’s correlation to assess the same and provided the correlation coefficients (r) along with their 95% CIs. A p-value < 0.05 was deemed statistically significant. HFpEF: Heart failure with preserved ejection fraction, HFmrEF: Heart failure with mid-range ejection fraction, HFrEF: Heart failure with reduced ejection fraction, LVEF: Left ventricular ejection fraction, ABI: Ankle-brachial index

Parameters	r (95% CI)	p-value
ABI with NT-proBNP		
Total (n = 801)	-0.059 (-0.129 to 0.009)	0.139
HFpEF (n = 166)	-0.149 (-0.294 to 0.004)	0.077
HFmrEF (n = 418)	-0.025 (-0.120 to 0.071)	0.359
HFrEF (n = 217)	-0.035 (-0.167 to 0.099)	0.774
ABI with LVEF		
Total (n = 801)	0.057 (-0.012 to 0.126)	0.293
HFpEF (n = 166)	0.164 (0.012 to 0.309)	0.015
HFmrEF (n = 418)	0.020 (-0.076 to 0.115)	0.458
HFrEF (n = 217)	0.014 (-0.119 to 0.147)	0.507

**Figure 10:**
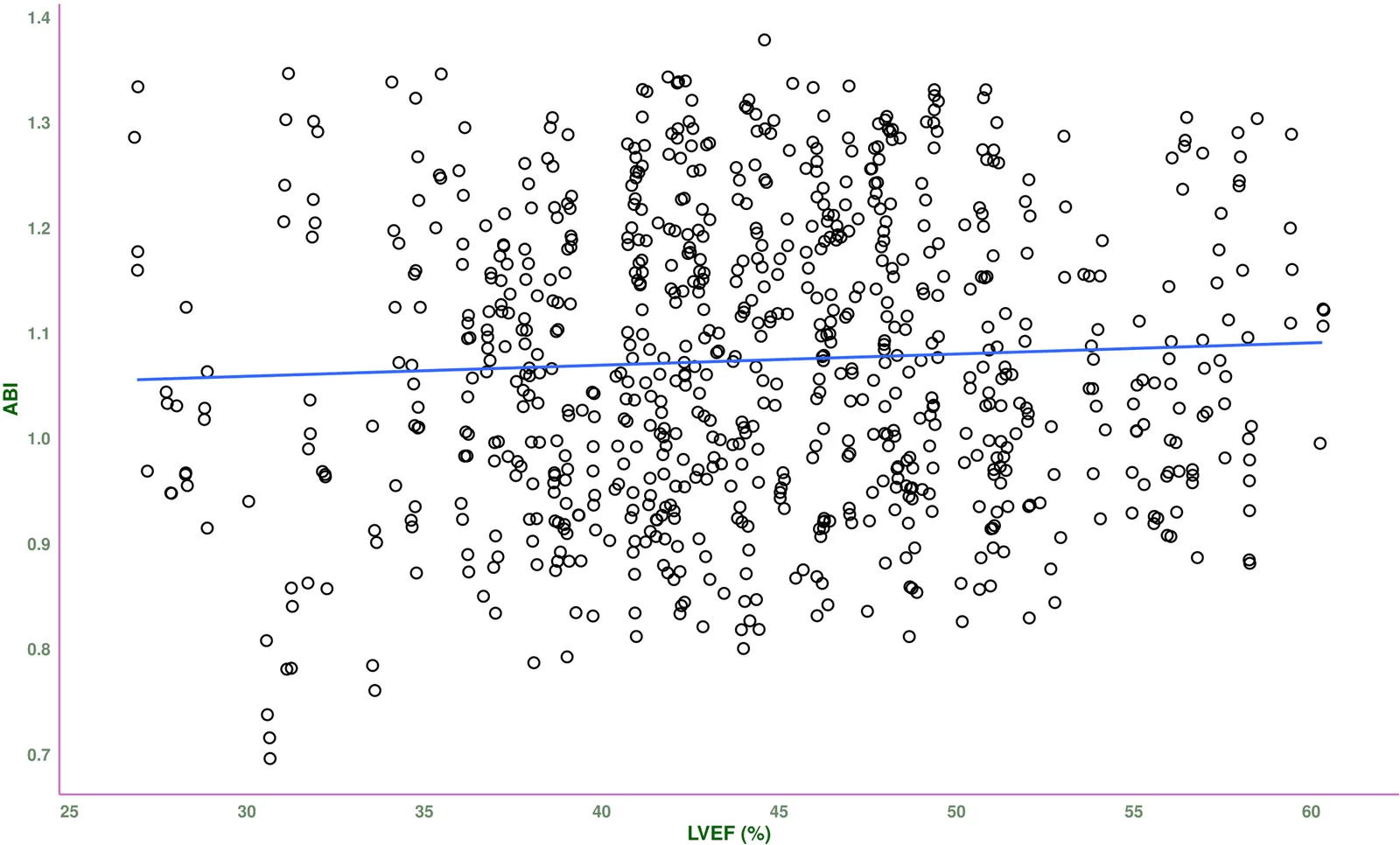
Correlation of ABI and LVEF The jitter plots illustrate the correlation between ABI and LVEF among participants. We used Spearman’s correlation to assess the same. A p-value < 0.05 was deemed statistically significant. The authors used R software version 4.6.1 (R Foundation for Statistical Computing, Vienna, AUT) [27] to generate this plot. ABI: Ankle-brachial index, LVEF: Left ventricular ejection fraction

Figure 10 and Table 4 demonstrate the correlation between ABI and LVEF among participants. There was a weak positive correlation (r = 0.057, 95% CI = -0.012 to 0.126, p = 0.293) between ABI and LVEF across all participants. The correlations were also positive among those with HFpEF (r = 0.164, 95% CI = 0.012 to 0.309, p = 0.015), HFmrEF (r = 0.020, 95% CI = -0.076 to 0.115, p = 0.458), and HFrEF (r = 0.014, 95% CI = -0.119 to 0.147, p = 0.507).

## Discussion

In this study, we evaluated and compared the glycemic parameters (i.e., FBS, PPBS, and HbA1c), ABI, NT-proBNP, and LVEF of the participants with HFpEF, HFmrEF, and HFrEF. We assessed the correlations of ABI with NT-pro BNP and LVEF in the participants. We did not observe any clinically significant differences in the glycemic parameters. The ABI values in participants with HFpEF were lower than in those with HFmrEF or HFrEF. Our observations were corroborated by the study by Chen et al. [23]. The differences in NT-proBNP in participants with different LVEF were both clinically and statistically significant. Our findings were consistent with studies by Özlek et al. [28] and Tromp et al. [29]. The negative correlation (though weak) between ABI and NT-pro BNP suggests that patients with reduced LVEF should be investigated for PAD. The positive correlation (though weak) between ABI and LVEF suggests that patients with preserved LVEF have a lower likelihood of vascular impairment of the lower limbs.

NT-proBNP in heart failure

The functional and structural integrity of the left ventricle modulates the plasma levels of NT-proBNP [16]. Internal ventricular dimensions, wall thickness, and interventricular pressure modulate the ventricular wall stress and cardiomyocyte stretch [30]. In HFpEF, the observed increase in LV dimensions is smaller than the increase in wall thickness [16,30]. The production of NT-proBNP is triggered by an increased wall tension, especially in early phases of HFpEF [15,16]. In such cases, the patient might show subdiagnostic NT-proBNP values [16,30]. Moreover, NT-proBNP levels vary with age and gender [13,14]. Their values tend to rise in elderly individuals and women [14]. Regardless of sex or the severity of heart failure, NT-proBNP levels almost double with every decade of age [14,18]. The diagnostic utility of NT-proBNP declines in individuals over 70 years with low renal function (eGFR <45 mL/min/1.73 m²) [18]. Nevertheless, prognostic accuracy remains constant across age groups, suggesting that elderly patients might benefit from higher cutoffs [18]. Januzzi et al. advocated the following age-specific NT-proBNP thresholds for heart failure: ≥ 450 pg/mL in individuals < 50 years, ≥ 900 pg/mL in those aged between 50 and 75 years, and ≥ 1800 pg/mL in those > 75 years [17].

ABI, PAD, and heart failure

Patients with heart failure and PAD often have other chronic diseases like T2DM and hypertension [21]. The risks of morbidity and mortality are higher among heart failure patients with PAD than those without PAD [20-22]. The ABI is a predictor of cardiovascular functional impairment irrespective of PAD symptoms [23]. Arteriosclerosis due to vascular calcification occurs earlier in the lower than in the upper limbs [21,23]. Vascular calcification triggers ventricular hypertrophy, increased cardiac afterload, cardiac diastolic dysfunction, decreased coronary artery perfusion, myocardial ischemia, and heart failure due to arterial stiffening [23]. Furthermore, it also increases the risk of cognitive decline and all-cause mortality [21,23]. This vascular calcification also causes hyperphosphatemia and uremia in renal failure patients [23].

Higher NT-proBNP levels indicate lowered myocardial contractility and reduced LVEF [16,19]. Lower ABI values indicate a higher likelihood of ischemic changes in lower limbs [22-24]. Our findings concatenated these statements through a negative correlation between ABI and NT-proBNP. We also found a weak positive correlation between ABI and LVEF. These findings suggest that individuals with a low LVEF, ABI, and high NT-proBNP values must be assessed for PAD symptoms.

Clinical implications

The participants with HFpEF generally had their ABI values within the normal range. However, the participants with HFrEF must be followed up to re-evaluate their ABI values. Though NT-proBNP is affected by advancing age, the participants with HFrEF tended to have higher NT-proBNP values than those with HFpEF. Hence, age stratification and corresponding thresholds must be considered while deciphering the NT-proBNP values of any patient with heart failure. The patients and their relatives must be educated and trained about the significance of heart failure symptoms, abnormal ABI, and reduced LVEF.

Strengths and limitations

The major plus in this study is the analysis of both ABI and NT-proBNP in participants with three types of heart failure. Some limitations were also present in our study. First, our findings cannot be generalized due to the strict criteria and the lack of follow-up data. Second, we could not correlate the echocardiography findings with ABI and NT-proBNP. Third, we did not assess the role of medications and comorbidities on NT-proBNP and ABI. Fourth, we could not perform age- and gender-stratification of NT-proBNP values, which could have helped establish a precise correlation between NT-proBNP and ABI. Fifth, we did not perform a multivariate analysis, which could have yielded more accurate results.

## Conclusions

Our study demonstrated that patients with T2DM and HFrEF are more prone to myocardial stress, systolic dysfunction, and PAD. The correlations of ABI with NT-proBNP and LVEF were either weakly positive or weakly negative. However, the findings suggest that peripheral vascular disease, increased cardiac strain, and compromised ventricular function could have a common pathophysiology. These results emphasize the potential of ABI as an easy, non-invasive screening method for determining the risk for cardiovascular disease. When combined with echocardiographic evaluation of LVEF and NT-proBNP measurement, ABI might enhance early identification, risk assessment, and treatment of heart failure in diabetic individuals. Therefore, combining vascular evaluation with cardiac biomarkers and imaging can offer a clear assessment of heart failure. We recommend prospective studies with longer follow-up periods and larger sample sizes to explore the diagnostic and prognostic roles of ABI and NT-proBNP in patients with heart failure.
